# An immune therapy model for effective treatment on inflammatory bowel disease

**DOI:** 10.1371/journal.pone.0238918

**Published:** 2020-09-24

**Authors:** Anna Park, Sangil Kim, Il Hyo Jung, Jong Hyuk Byun

**Affiliations:** 1 Department of Mathematics, Pusan National University, Geumjeong-Gu, Busan, South Korea; 2 Finance·Fishery·Manufacture Industrial Mathematics Center on Big Data, Pusan National University, Busan, South Korea; 3 Institute of Mathematical Sciences, Pusan National University, Busan, South Korea; Biotechnology HPC Software Applications Institute (BHSAI), UNITED STATES

## Abstract

Inflammatory bowel disease (IBD) is a disease that causes inflammation throughout the digestive tract. Repeated inflammation and frequent relapses cause intestinal damage and expose the patient to a higher risk. In this work, we proposed an immune therapy model for effective treatment strategy through mathematical modeling for patients with IBD. We evaluated the ability of the patient’s immune system to recover during treatment. For this, we defined the interval of healthy individual, and examined the frequency of compartments such as T cells and cytokines considered in the model maintain the normal state. Based on the fact that each patient has a unique immune system, we have shown at the same drug works differently, depending on the individual immune system characteristics for every patient. It is known that IBD is related to an imbalance between pro- and anti- inflammatory cytokines as the cause of the disease. So the ratios of pro- to anti- inflammatory cytokines are used as an indicator of patient’s condition and inflammation status in various diseases. We compared the ratios of pro- to anti- inflammatory cytokine according to patient’s individual immune system and drugs. Since the effects of biological drugs are highly dependent on the patient’s own immune system, it is essential to define the immune system status before selecting and using a biological drug.

## Introduction

IBD causes inflammation in the digestive system and is characterized by a disproportion of cytokines in the affected patients [1, 2]. Two major types of cytokines are known, pro- and anti- inflammatory cytokines [3]. Pro-inflammatory cytokines, such as tumor necrosis factor (TNF-*α*), interleukin 1 (IL-1), and interleukin 6 (IL-6), produce inflammation, whereas anti-inflammatory cytokines such as interleukin 4 (IL-4) and interleukin 10 (IL-10) reduce inflammation [3, 4]. In patients with IBD, pro-inflammatory cytokine levels are higher and anti-inflammatory cytokine levels are lower than those in healthy individuals. During the disease, inflammatory processes lead to increased pro-inflammatory cytokine levels, mediated by a T cell response.

Currently, treatment methods involve acquiring control of cytokine levels in the immune system for patients with IBD. Pro-inflammatory cytokines are found to be involved in T cell and monocyte signaling, and control of pro-inflammatory cytokines demonstrated the effect of reducing T cell activation by inducing T cell apoptosis [5].

Anti-TNF-*α* was first used for IBD in doses similar to those of infliximab, adalimumab, and certolizumab [6–8]. Tumor necrosis factor (TNF) is a protein produced in the body by macrophages and T lymphocytes. The expression of TNF-*α* is increased in the intestinal mucosa in IBD patients. This suggests that antibody neutralization studies for TNF-*α* play an important role in understanding the pathogenesis of IBD.

Besides TNF-*α*, other pro-inflammatory cytokines have been developed to target IL-12/IL-23, IFN-*γ* and IL-6R in order to inhibit immune system activation [9–11]. IL-12/IL-23, IFN-*γ*, and IL-6R are the highly-expressed pro-inflammatory cytokines within the inflamed mucosa of IBD patients [12]. Thus, therapy strategies targeting these proteins, have been effective and are being currently applied to these patients. These therapies are based on antibody-mediated inhibition of cytokines, preventing them from increasing continually.

Another treatment option utilizes injection of anti-inflammatory cytokines to counterbalance the levels of pro-inflammatory cytokines. Anti-inflammatory cytokines such as IL-10, 11, and IFN-*γ*, have been developed as biological treatment candidates for IBD. Treatment involves injection of a high dose of IL-10 to maintain a sufficient level for repair of the damaged intestinal mucosa [13–15]. Patients with chronic inflammatory diseases and fast recovery show high serum levels of IL-10 [16, 17].

Meanwhile, there are mathematical models related to IBD. The focus of many mathematical models is the observation of an abnormal immune system. In [18], abnormal levels of helper T cells (Type 1 T helper cell and Type 2 T helper cell) are observed by regulatory T cells in IBD. Jansen et al. [19] explored the relationship between the production of cytokines and immune deficiency and the role of macrophages in the development and progression of IBD through a mathematical model to induce IBD pathophysiology.

Patients with IBD have symptoms of recurrent inflammation and rupture of the digestive tract. Without timely proper treatment, it is difficult to recover damaged tissue from persistent inflammation. The order of treatment for IBD has not been studied; however, there is a report on the effect of treatment regimen using a given drug in the therapy of cancer [20]. Combining treatment with a mathematical model based on the abnormal immune response of IBD, we develop an immune therapy model for suggesting the order of treatment for IBD. Although a variety of cytokine-targeted therapies are used for IBD, our study is important because there is no guidance on the order of these suggested treatments.

Here, we report the application of mathematical modeling for observation of the patient’s immune system during each treatment. We classified the patient’s immune system into four types and defined the interval of healthy individuals by evaluating the ability of the patient’s immune system to recover during treatment. We compared the ratios of pro- to anti- inflammatory cytokine according to a patient’s individual immune system and drugs used. The ratios of pro- to anti- inflammatory cytokines are used as an indicator of the patient’s condition and inflammation status from various diseases. The effect of the treatment was considered to be the degree of recovery from an upset immune system. In parallel, the immune system of other patients under the same conditions reacted differently to the therapeutic drug. These results demonstrate that the choice of a particular drug depends on the unique individual immune system. It is, therefore, essential to observe the patient’s own immune system at the start of treatment.

To prove the above-mentioned suggestions, we developed a mathematical model involving drug injection (described in Materials and methods) and performed its subsequent simulation and analysis (Results).

## Materials and methods

We developed a mathematical model to show the dynamics of the immune system of patients with Crohn’s disease upon receiving biological agents. Understanding the relationship between the patient’s immune system and biological agents from this mathematical model is important for the treatment of patients. The mathematical model is based on the study of Lo et al. [21]. In [21], the differential equation system was developed based on a network that integrates cytokines and transcription factors associated with Type 1 T helper cell(Th1), Type 2 T helper cell(Th2), Type 17 T helper cell(Th17) and regulatory T cell pathways. Our model is substituted with an additional compartment for drug intervention.

### Blockade of pro-inflammatory cytokine therapy

Crohn’s disease is characterized by high levels of pro-inflammatory cytokines in the body. High levels of pro-inflammatory cytokines stimulate inflammation and further worsen the symptoms of the disease. Since the reasons and mechanisms of sustained high levels of pro-inflammatory cytokines in IBD are unknown, the initial treatment includes inhibition of the activity of continuously produced pro-inflammatory cytokines.

The mechanism of the treatment for blocking pro-inflammatory cytokines is neutralization of target cytokines by inactivating them with targeted antibodies injected into the body. Based on this mechanism, we developed the following equation, describing drug intervention-blockade of pro-inflammatory cytokine therapy.

$$\begin{array}{cc} \frac{dD_{p}}{dt} & =v_{M}\left(t\right)-\sigma ID_{p}-\delta D_{p},\\ v_{M}\left(t\right) & =\{\begin{array}{c} \text{constant},\;t=\text{given}\;\text{time}\;\text{period},\\ 0,\;\;\;\text{otherwise.} \end{array} \end{array}$$

*D*_*p*_ refers to the antibody concentration of pro-inflammatory cytokine in the body. *v*_*M*_(*t*) is the concentration of drug, injected over a given time period according to the assumed therapeutic method. The injected antibody binds to the target cytokine to neutralize its activity (represented by the term *σID*_*p*_). *I* represents target pro-inflammatory cytokine. The rate of neutralization of the target cytokine by binding of the drug is represented by *σ*. *δ* represents the rate at which the antibody fails to bind the target cytokine and is excreted from the organism. *σ* and *δ* are characteristic parameters indicating the effect of the drug.

#### Anti-TNF-*α* therapy

Currently, the most representative treatment is the use of a biological agent that inhibits one of the major pro-inflammatory cytokines, TNF-*α*. Increased levels of TNF-*α* in the mucosa of Crohn’s disease patients were reported. Thus, the earliest developed therapy is one that inhibits TNF-*α* and generally, this is the first biological agent tried in patients.

The cytokine was named TNF because of the observation that injection of bacterial cultures into cancer patients induces tumor necrosis [22]. TNF not only eliminates cancer cells but also affects normal cells and acts as a cofactor for phagocytosis, activation of T cells, and antibody production by the B cells. However, abnormalities such as excess of TNF can lead to inflammation, allergies and cancer. In patients with Crohn’s disease, chronic inflammation, caused by excess of TNF, results in repeated cell destruction and recovery, leading to cell fibrosis, which in turn, causes complications such as tissue hardening and eating disorders. Therefore, to prevent excess TNF, anti-TNF-*α* is injected to prevent TNF-*α* activation [23]. It inhibits TNF-*α*-induced inflammatory cytokine induction, increases leukocyte migration, activation of neutrophils and eosinophils, and induction of acute reactants in the liver. Thus, it inhibits the binding of TNF-*α* to the p55 and p75 TNF receptors and lyses surface TNF-*α*-expressing cells in the presence of complement. Thus, depletion of TNF-*α* and abrogation of TNF-*α*-mediated inflammatory signal occur. This treatment leads to the inhibition of inflammation and cell destruction in the intestinal mucosa.

$$\begin{array}{ccc} \frac{dI_{\alpha }}{dt} & = & v_{\alpha M}M_{1}+v_{\alpha 1}T_{1}-\delta_{\alpha }I_{\alpha }-\sigma_{D\alpha }I_{\alpha }D_{\alpha },\\ \frac{dD_{\alpha }}{dt} & = & v_{M}-\sigma_{D\alpha }I_{\alpha }D_{\alpha }-\delta_{D\alpha }D_{\alpha }. \end{array}$$

*I*_*α*_ indicates the concentration of TNF-*α*. TNF-*α* is produced by *M*_1_ macrophage and Th1 cells. It decays along with the passing time. *D*_*α*_ is a concentration of anti-TNF-*α*. *σ*_*Dα*_ *I*_*α*_ *D*_*α*_ is neutralized rate and we give some value *δ*_*Dα*_in equations *I*_*α*_ and *D*_*α*_. The injection of the drugs is represented by *v*_*M*_ = *v*_*M*_(*t*). *δ*_*Dα*_ is a degradation rate of anti-TNF-*α*.

#### Anti-IL-12/IL-23 therapy

Anti-IL-12/IL-23 Therapy is to inject fully human IgG1-*κ* monoclonal antibody into the body. It is based on the theory that abnormal regulation of IL-12 and IL-23 in the body causes IBD [24]. IL-12 and IL-23 are biological agents developed for inhibiting disease activity by blocking the interaction of IL-12R*β*1 receptor protein on the cell surface [25, 26]. It is usually the next biological agent that administered to patients who do not demonstrate an effect of anti-TNF-*α*. Our study is based on the assumption that only IL-12 is inhibited. The model is as follows:

$$\begin{array}{l} \frac{dI_{12}}{dt}=v_{12M}\frac{M_{1}}{1+I_{10}/\zeta_{10}}-\delta_{12}I_{12}-\sigma_{D12}I_{12}D_{12},\\ \frac{dD_{12}}{dt}=v_{M}-\sigma_{D12}I_{12}D_{12}-\delta_{D12}D_{12}. \end{array}$$ (1)

*I*_12_ is the concentration of IL-12. IL-12 is produced by M1 macrophage and is suppressed by IL-10. *D*_12_ is the concentration of anti-IL-12. *σ*_*D*12_ *I*_12_ *D*_12_ is the neutralization rate and we give some value *σ*_*D*12_ in equations *I*_12_ and *D*_12_. *δ*_*D*12_ is degradation rate of anti-IL-12. Table 1 presents the parameters for the systems (1).

**Table 1:** Parameters used in the systems of blockade of pro-inflammatory cytokine therapy.

Parameter	Definition
*v*_*αM*_	Production rate of TNF-*α* by macrophages
*v*_*α*1_	Production rate of TNF-*α* by Th1
*δ*_*α*_	Degradation rate of TNF-*α*
*σ*_*Dα*_	Rate at which *I*_*α*_ is neutralized by *D*_*α*_
*v*_*M*_	Injection of the drugs
*δ*_*Dα*_	Degradation rate of anti-TNF-*α*
*v*_12*M*_	Production rate of IL-12 by macrophages
*ζ*_10_	Half-saturation constant for IL-10
*δ*_12_	Degradation rate of IL-12
*σ*_*D*12_	Rate at which IL-12 is neutralized by *D*_12_
*δ*_*D*12_	Degradation rate of anti-IL-12

### Administration of anti-inflammatory cytokine therapy

Imbalances between Th1 and Th2 immune responses are observed in Crohn’s disease, which lead to a deregulated balance between pro-inflammatory and anti-inflammatory cytokines [3, 27]. Thus, patients with Crohn’s disease are characterized by low levels of anti-inflammatory cytokines [28]. Anti-inflammatory cytokines play an important role in treating inflammation and restoring immunity. Treatment with anti-inflammatory cytokines restores the insufficient amounts of anti-inflammatory cytokines in the organism. In this case, human recombinant anti-inflammatory cytokine proteins are used. Thus, the patient’s own immune system is activated for treatment of the disease from within, which is called autoimmune therapy. Utilizing their own defense ability, the patients do not develop side effects, making this type of the therapy advantageous in many aspects. Therapies(blockade of pro-inflammatory cytokines therapy) that artificially suppress immune responses are contrary to the consequences of side effects from immune deficiency, such as tuberculosis and allergies. Injection of anti-inflammatory cytokines has not been commercialized. However, there are ongoing clinical trials, aiming for prevention of the progress of inflammation by counterbalancing pro-inflammatory cytokines with the anti-inflammatory cytokines [29]. In our study, we simulated a feasible treatment through using our model.

The formula for this cure is clearly straightforward. *D*_*A*_ is the concentration of the injected recombinant anti-inflammatory cytokine. *v*_*M*_(*t*) is the concentration of the drug, injected over a given time period according to the assumed treatment regimen. *δ* represents the rate, at which recombinant anti-inflammatory cytokine does not play a role and is excreted from the organism. This formula differs from the pro-inflammatory cytokine antibody treatment, because it is injected with insufficient amount of cytokines.

$$\begin{array}{c} \frac{dD_{A}}{dt}=v_{M}\left(t\right)-\delta D_{A}. \end{array}$$

#### IL-10 therapy

IL-10 is one of the representative anti-inflammatory cytokines. It is an inhibitory cytokine, important for induction or maintenance of immune tolerance [30]. In 1993, Kuhn et al reported that spontaneous colitis occurs naturally in mice lacking IL-10 gene, which has been used as a representative model of IBD [31]. This finding suggests a connection with Crohn’s disease. In a clinical trial involving 46 patients with steroid-induced Crohn’s disease, only 23% of the patients in the placebo group had developed, whereas 50% of the IL-10 infusions had developed. However, in later large-scale clinical trials, despite clinical improvement, no remission was observed [32, 33]. The drug is still in clinical trials and several studies have shown its effect in treating Crohn’s disease.

Therefore, we developed the following equation. *I*_10_ means the concentration of IL-10. IL-10 is produced by M2 macrophages and Treg cells, IFN-*γ* aids and IL-2 interferes with this production. *D*_10_ means the concentration of injected recombinant IL-10. In particular, it was not assumed that recombinant IL-10 would be injected into the body so that all sheep would act as the role of IL-10. It was assumed that it would play the role of an anti-inflammatory cytokine in the body as much as the ratio of *v*_*D*10_. The parameter *v*_*D*10_ is an important factor indicating the effect of medicine. Table 2 presents the parameters for the system (2).

$$\begin{array}{l} \frac{dI_{10}}{dt}=v_{10M}M_{2}+v_{10r}-[1+n_{2r}(\frac{I_{\gamma }}{\zeta_{2}+I_{2}})]T_{r}\delta_{10}I_{10}+v_{D10}D_{10},\\ \frac{dD_{10}}{dt}=v_{M}-\delta_{D10}D_{10}. \end{array}$$ (2)

**Table 2:** Parameters used in the system (2) of administration of anti-inflammatory cytokine therapy.

Parameter	Definition
*v*_10*M*_	Production rate of IL-10 by macrophages
*v*_10*r*_	Production rate of IL-10 by Treg
*n*_2*r*_	Production rate of IL-10 by IL-2
*δ*_10_	Degradation rate of IL-10
*v*_*D*10_	Rate of drug acting as IL-10
*v*_*M*_	Injection of the drugs
*δ*_*D*10_	Degradation rate of anti-IL-10

## Results

The purpose of mathematical modeling is to observe changes in the patient’s overall immune system during treatment. Currently used biologic therapies inhibit one type of the highly expressed pro-inflammatory cytokines. However, not much attention is paid to the changes in the patient’s immune system resulting from the inhibition of over-produced cytokine activity. The results of simulations through mathematical models suggest that observation of changes in the immune system is an essential process for the treatment. Assuming that the parameters of the patient’s unique immune cells remain unchanged, alterations in the immune system are examined through the treatment.

In consideration of the characteristics of patients with IBD, [21] compared patients with Crohn’s disease to a normal intestinal mucosa. Lo et al. [21] also examined the activities of Th1 and Th2 with respect to the normal levels and classified them into four categories. Of the four categories, the first involved Th1 levels being higher and Th2 levels being lower than in healthy individuals. However, category two showed the reverse effect, with lower levels of Th1 and higher levels of Th2. Case 3 involved both Th1 and Th2 levels being higher than in healthy individuals, whereas case four revealed lower levels of Th1 and Th2 in comparison to those in healthy individuals. Parameters are defined for each of the four cases. Ten parameters showed distinctive values for each case. In Case 3, the coefficient of IFN-*γ* was increased by 194% of that in normal subjects and was lower than that of normal subjects IFN-*γ*. For TNF-*α*, the most essential observation, 11% showed the highest parameter value in case 4 and the lowest in case 1. It is shown that the helper T cell activity of the partial mesangial membrane is not related to the cytokine production rate.

**Fig 1:**
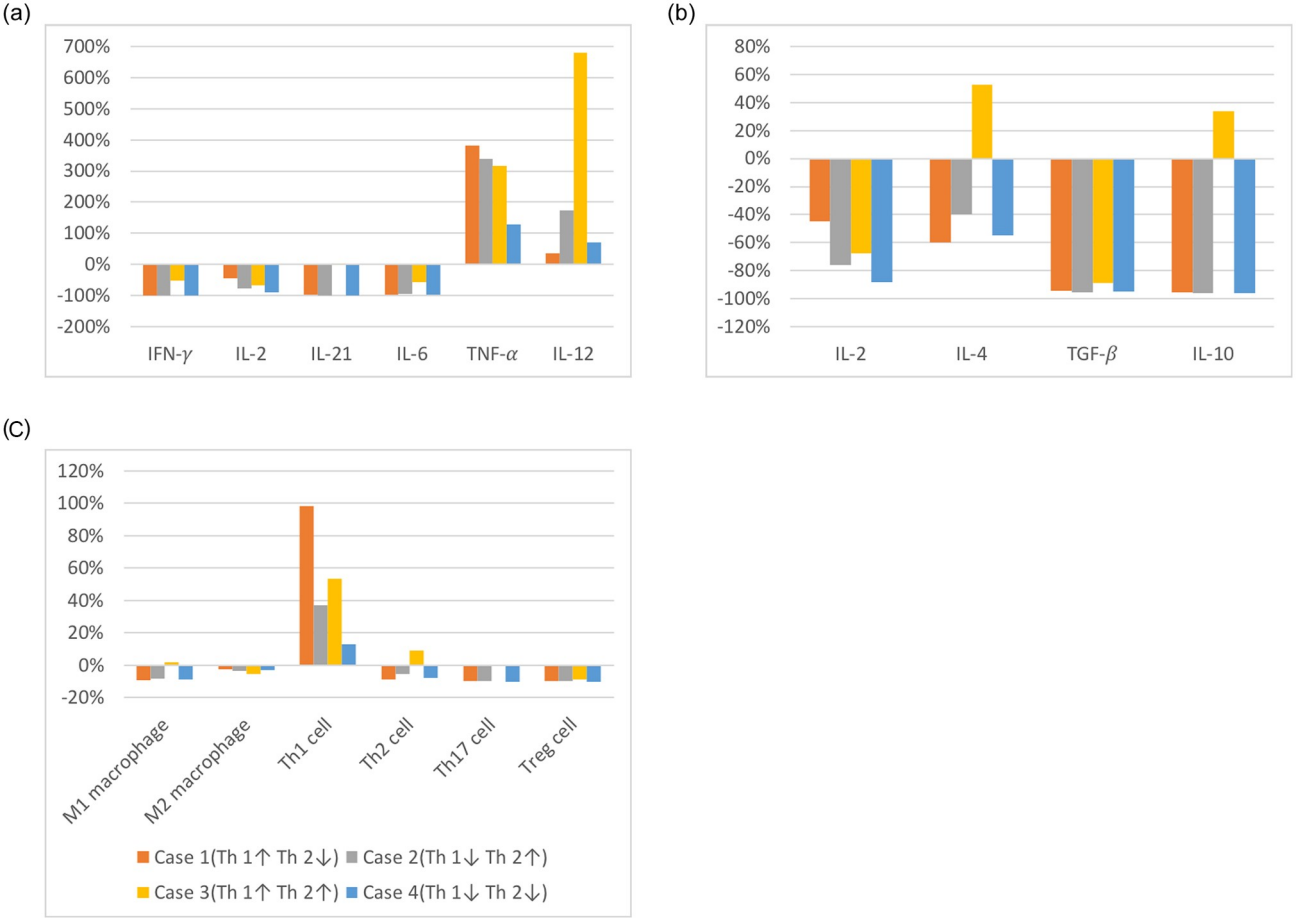
Fold changes of the cytokine and T cell concentrations from [21] in four cases of diseases. Orange bars represent case 1 of diseases; gray bars represent case 2 of diseases; yellow bars represent case 3 of diseases; blue bars represent case 4 of diseases; (a) pro-inflammatory cytokines group, (b) anti-inflammatory cytokines group, (c) immune cells group.

Fig 1 shows the changes in the steady-state concentrations in each case, based on the level of healthy individuals. Since each case has different parameters, they all show different steady-state concentrations. Fig 1 shows the exact characteristics of Crohn’s disease. Concentrations of TNF-*α* and Th1 were higher than in the general population in all cases. Except in case 3, anti-inflammatory cytokine concentrations were lower than that in the general population. Anti-inflammatory cytokines were consistently low, but in case 3, IL-4 and IL-10 were higher than normal. Based on these results, the focus of this simulation is dynamics of the steady-state concentrations after the treatment.

### Blockade of pro-inflammatory cytokine therapies

Next, we applied commercially available anti-pro-inflammatory cytokines, anti-TNF-*α* and anti-IL-12, to simulate and analyze the model. Both treatment strategies followed the same treatment with infliximab. Infliximab is injected at 0, 2, 4, and 8-week intervals, followed by an intravenous injection at 8-week intervals. The dosage injected into the patient depends on the patient’s weight. Therefore, on the basis of the paper, the levels of infusion concentrations of anti-TNF-*α* and anti-IL-12 were determined based on the concentration of the drug remaining in the patient’s body after injection.

The effective therapy aims to decrease the levels of pro-inflammatory cytokines that are higher than normal, and increase the levels of anti-inflammatory cytokines that are lower than normal. Similarly, the levels of the helper T cells also have the same effect. However, it is not easy to assess whether different patients have different concentrations of pro-inflammatory and anti-inflammatory cytokines. There are no appropriate standards or guidelines for such an assessment. Therefore, various methods were used to analyze the status of immune system during treatment.

#### Interval of healthy individual

During treatment, the immune system attempts draws oscillation to achieve homeostasis of immunity. Because patients have a imbalanced immune system, it is common for the immune system to lower the amount of cytokines produced at the time of injecting an anti-body and then gradually recover during the next eight weeks of injection. At this time, a change occurs in the immune system by reducing the amount of the target cytokines. Nevertheless, other cytokines are affected by each other due to the mutual cross-talk regulation. Therefore, we define the normal level interval and evaluate whether the patient’s T cells and cytokines stay within the normal level. The normal level interval is represented as follows.

$$\begin{array}{c} \text{interval}=\pm |\frac{\text{level}\;\text{of}\;\text{patient}-\text{level}\;\text{of}\;\text{healthy}\;\text{indiviual}}{2}|. \end{array}$$ (3)

**Fig 2:**
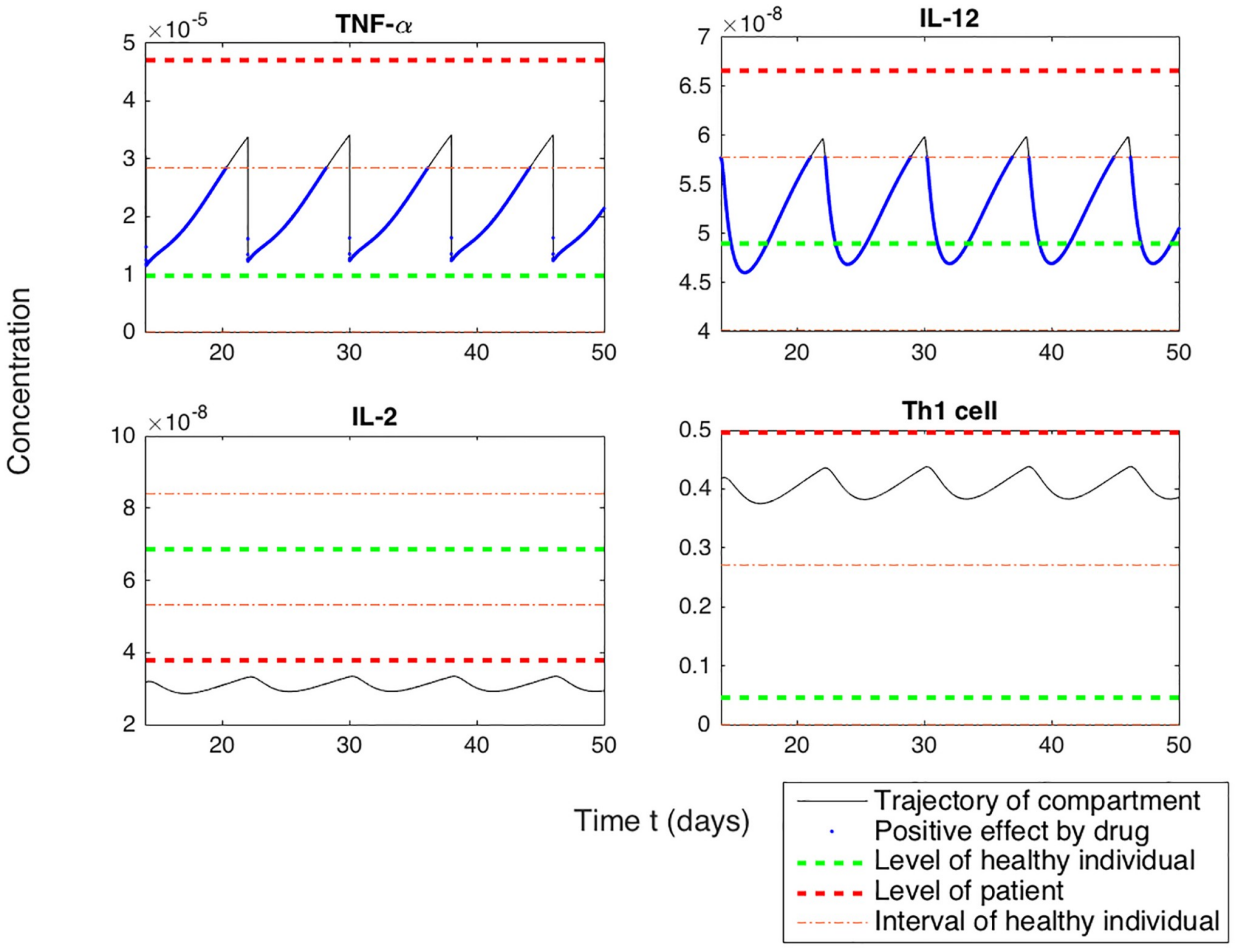
The frequency of immune cells continuing at a healthy interval(50%) during the treatment of anti TNF-*α* in case 1. The time series of TNF-*α*, IL-12, IL-2 and Th1 cell concentrations(*g*/*cm*^3^) depicted during the treatment of anti TNF-*α*. The solid line depicts the trajectory of the given compartment. The red dotted line shows the patient’s level and the green dotted line shows the level of a healthy individual’s. The interval of a healthy individual is defined that normal level was added and subtracted half of the difference between the patient and the normal level(50%). This was marked with an orange dotted line. The points entering the interval were defined as a positive effect of drug, and were shown as a blue dotted line.

**Fig 3:**
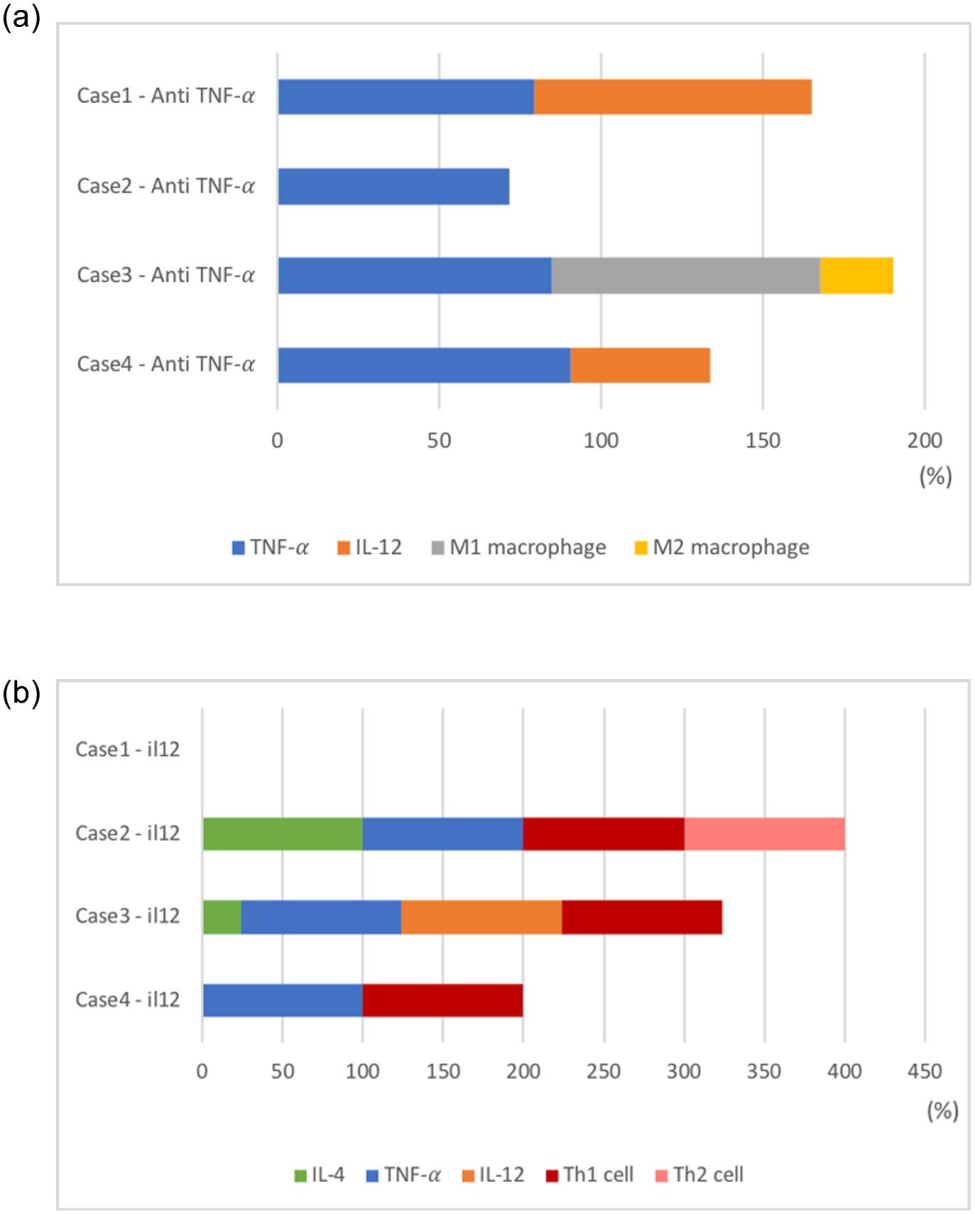
The frequency of immune cells continuing at a healthy interval. As shown in Fig 2, the positive effects by drug(blue dotted line) are expressed as a percentage for all compartments in each case of diseases. The stacked bar graphs represent the frequency staying at the healthy interval in each cases of disease; (a) in treatment of anti-TNF-*α*, (b) in treatment of anti-IL-12.

The purpose of this simulation was to evaluate the ability and frequency of the patient’s immune system to recover and maintain the normal state during the treatment. When the treatment period was set to 50 weeks, we analyzed how long a patient was in the normal level interval for 14 to 50 weeks. Since the initial 13 weeks were influenced by the initial value, the results were reflected from the 14th week after the start of the injection treatment. There is a marked difference in each case. When the treatment is applied, if the patient’s level stays within the normal level interval at all times, it is defined to be 100%. In case 1, for a given time applied anti-TNF-*α* therapy, TNF-*α* stay 79.59% and IL-12 stay 85.26% (Fig 2(a) and 2(b)). Other compartments do not stay in normal level interval (Fig 2(c) and 2(d)). In case 2, only TNF-*α* stays in the normal interval(anti-TNF-*α* therapy), but Th1 and Th2 stays in the interval applied anti-IL-12 therapy (Fig 3). Since this study is not based on experimental evidence, we cannot claim that some drugs are more effective, but we can see that under the same conditions, the overall immune system condition can change depending on the drug. Knowing the relationship between these changes and the effect of the drug can be an opportunity to increase the frequency of desired outcomes for patients.

#### Ratios of pro- to anti- inflammatory cytokines factors

Immune tolerance is discussed as one of the causes of autoimmune diseases. This is because the ratios of pro- to anti- inflammatory factors balance is disturbed due to the high pro-inflammatory levels and low anti-inflammatory levels as the matter of the disease. The balance between pro-inflammatory and anti-inflammatory factors determines the incidence and consequences of inflammation. The imbalance between these factors is the onset and catalysator of the inflammation, which additionally can indirectly affect the ratio of helper T cells to T regulatory cells. Eventually, it affects the progression of inflammation and increases inflammation [3, 4, 6–8, 21]. Thus, in various immune-related diseases, the focus is on the ratio of pro-inflammatory and anti-inflammatory cytokines [34–36]. Therefore, it is important to observe whether the already disturbed immune tolerance returns to homeostasis through treatment. S1A Table in S1 File shows the pro- to anti- inflammatory factors ratios of patients in each case before treatment. The rate of change was expressed as a percentage, based on the ratios of healthy subjects. The ratios of TNF-*α*, IL-12, and Th1 are extremely high, compared to that in healthy individuals, and the levels of anti-inflammatory cytokines are low. Thus, it can be predicted that therapies targeting TNF-*α* and IL-12 can be used at the beginning. IL-12 directly, and TNF-*α* indirectly affect Th1 production through macrophages. Therefore, it is predictable through the model that these factors and eventually helper T cells can be controlled. It is also important to know how to control the factors to restore balance against anti-inflammatory factors.

S1B-S1E Table in S1 File show the change of ratios during anti-TNF-*α* and anti-IL-12 treatment in each case. The maximum and minimum values are indicated because of the natural changes within the immune system of the patient. The mechanism did not show unusually high or low values.

The ratios containing cytokines as targets are significantly reduced after the injection. In particular, in cases 1, 2 and 4, after treatment with anti-IL-12, not only the ratios of IL-12 to anti- inflammatory factors but also the values of the ratios TNF-*α* to anti- inflammatory factors were closer to the normal levels than those with anti-TNF-*α*. Although a stable normal ratio was not maintained, it appears to move from a pro-inflammatory state to an anti-inflammatory state during the treatment cycle. IL-12 and Th1 are closely related, and IL-12’s control eventually influences TNF-*α*, which is closely related to Th1. It also disproves that indirect inhibitory therapy may be more effective than direct therapy, depending on the characteristic parameters of the patient.

The amount of TNF-*α* is higher than normal in cases 1, 2, and 3, but the ratios of TNF-*α* to anti- inflammatory factors was lower within case 3 than cases 1 and 2. The maximum amount of anti-inflammatory cytokines was found in case 3, so the ratio was close to zero. By comparison, the ratios of IL-12 to anti- inflammatory factors is the largest in case 3. Case 3 is characterized by the largest amount of IL-12 and a lower amount of anti-inflammatory cytokines, resulting in greater balance. This information can be used to infer which drug should be applied initially in each case.

To consider only the behavior of the target cytokine is not sufficient. There are many reports on the importance of Th1/Th2 balance and its relationship with various diseases. However, in case 3, the anti-TNF-*α* treatment at Th1/Th2 ratio maintains the pro-inflammatory state, but does not deviate significantly from the healthy ratio. In this case, the Th1/Treg ratio maintains the pro-inflammatory state for both treatments, but the values are essential factors for sustained anti-inflammatory state. Consideration of the appropriate treatment should be prioritized based on the patient’s condition.

Recently, the importance of Treg as well as the importance of Th2 has been suggested [37]. Regulatory T cells(Treg cells) belong to the unique subpopulation of CD4 + T cells that play a pivotal role in maintaining immune tolerance and preventing autoimmunity to self-antigens. It is also part of a system that controls and balances the body’s immune response, preventing its overexpression and initiation of autoimmune diseases. Through these papers, Th1/Treg ratio will also play an important role in immune system analysis.

### Administration of anti-inflammatory cytokines

Patients with IBD are characterized by high levels of pro-inflammatory factors and low levels of anti-inflammatory cytokines. In previous section, we analyzed therapies that inhibit the pro-inflammatory cytokines, TNF-*α* and IL-12. In this section, we will analyze the treatment of patients with low cytokine levels via the injection anti-inflammatory cytokines in order to achieve normal cytokine levels. IL-10, for example, has been the focus of many studies and is currently used in treatment [31, 38]. Modeling and simulations were used to determine the dose to be used for pro-inflammatory cytokine therapy. We assumed that the dose obtained through these methods is accurate for achieving normal levels. The treatment cycle is the same as that for infleximab.

#### Analysis

**Fig 4:**
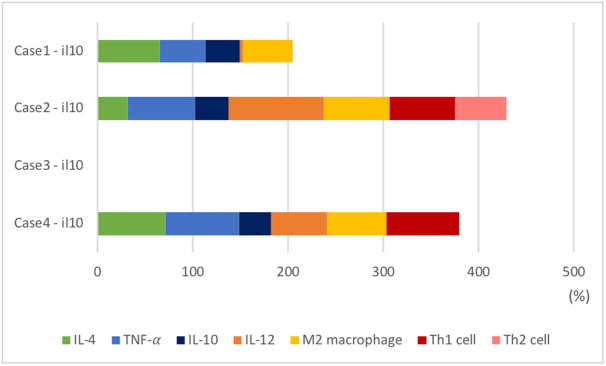
The frequency of immune cells maintained at healthy levels during administration of IL-10. As shown in Fig 2, the positive effects by drug(blue dotted line) are expressed as a percentage for all compartments in all cases of diseases. The stacked bar graphs represent the frequency staying the healthy interval each cases of disease in administration of IL-10.

The injection of IL-10 results in the production of a much wider range of immune factors, compared with that observed post the injection of anti-pro-inflammatory cytokines(Fig 4). IL-10 injection directly increases the levels of IL-12, macrophages, and Tregs and decreases the levels of Th1. In addition, IL-10 indirectly maintains IL-4, TNF-*α*, and Th2 at normal intervals. Case 2 shows that the largest number of immune cells are maintained at intervals over long periods of time. The results suggest that anti-inflammatory cytokine treatments are more effective in altering the overall immune system. In case 3, however, anti-inflammatory cytokine injection did not appear to significantly affect the immune system. The characteristics of the parameters in each case show different results even with the same treatment. This observation further supports the strategy of evaluation of the patient’s characteristics before treatment.

#### Ratios of pro- to anti- inflammatory cytokines factors

As with the analysis of anti-pro-inflammatory therapy, we analyzed the ratios of pro- to anti- inflammatory factors during therapy with IL-10, a deficient anti-inflammatory cytokine. Injection of IL-10 caused the balance between the ratios of TNF-*α* to anti- inflammatory factors and the ratios of IL-12 to anti- inflammatory factors to be closer to normal in treated patients than in IBD patients. However, in case 3, the ratio is more commonly at a pro-inflammatory state than that in cases 1, 2, and 4. IL-10 addition does not recover the ratios in all cases to normal levels (S1F Table in S1 File). It is not easy to compare treatments that block pro-inflammatory cytokines and those that involve injection of anti-inflammatory cytokines. However, according to our study, treatments that inhibit pro-inflammatory cytokines have many immunological side effects, whereas anti-inflammatory therapies are relatively safer [39]. Theoretically, anti-inflammatory therapy is expected to be as effective as pro-inflammatory therapy or even more effective than pro-inflammatory therapy in some patients.

## Discussion

IBD is one of the many autoimmune diseases, but its cause still remains unknown. Since the mechanisms of the onset of this disease are unclear, the development and use of therapy for this disease have been based on the patient’s condition. Patients with IBD have higher levels of pro-inflammatory cytokines and lower levels of anti-inflammatory cytokines than healthy individuals.

Cytokines are signaling molecules involved in the regulation of an individual’s immune response. They also play an important role in the development of immune cells and cell differentiation and regulation. Some cytokines amplify the immune response, while others suppress it. Thus, the malfunction of cytokine production and activity seems to play a central role in the cause and development of autoimmune diseases.

Therefore, the treatment strategy should be focused on the suppression of factors showing increased levels and complementing those showing lower levels, compared to the levels of these factors in healthy individuals. The latter is still a clinical treatment, while a variety of targeted pro-inflammatory cytokine therapies have been developed to address the former issue and have been used as a top priority for treatment.

In this paper, the model was developed by adding drug input equations based on the model reported by Lo et al. [21]. Four patients with IBD were categorized on the basis of their own parameters, and the changes in the immune system were examined along with the treatment response in these groups. Two treatments targeting TNF-*α* and IL-12 were modeled for the treatment of pro-inflammatory cytokines. It is not appropriate to form a judgement of the patient’s condition based on the levels of only one cytokine or immune cell. Moreover, we cannot definitely declare that this treatment is better or worse for the patient. The goal of treatment is to bring and maintain the levels of immune cells that are above or below normal levels to normal levels. Thus, an interval of a healthy individual was defined as the means for recovering and maintaining the normal levels during treatment. We also analyzed the ratio between the levels of pro-inflammatory and anti-inflammatory factors. The aim of this analysis was to define the impact of the balance between the pro-inflammatory and immunosuppressive functions of cytokines to the pathogenesis of autoimmune diseases. In many diseases, the ratios of pro- to anti inflammatory cytokines are used as an indicator of the patient’s condition and as an indicator of inflammation improvement.

We used a mathematical model to observe the recovery of the disturbed the ratios of pro- to anti- inflammatory cytokines balance during treatment. Four cases, based on the activity of helper T cell types 1 and 2, were considered. Patients with the same conditions were compared by simulations for different treatments. Our results were quite different and showed that selecting a drug after analyzing the patient’s native immune system can be more effective in treating the disease. In this study, there are obvious limitations in the inability to use actual data and the inability to express all various individual immune systems. However, the main advantage of the mathematical model is that it is possible to briefly express and understand the phenomenon of complex mechanisms. We applied compartments that are the most characteristic of patients with IBD, and it is believed that the patient’s tendency is expressed. Starting with our model, studies are possible that include extended immune systems such as Th17 cytokines (IL-17, IL-21, IL-22) and B-cell pathway.

The primary motivation for this study was to determine which medications could be more effective for a particular patient at the initial stage of treatment. However, it is very difficult and dangerous to conclude that certain drugs work better than others. Therefore, we have explained in this S1 File and identified the key beneficial drug for the patient, according to the specific characteristics of the patient’s immune system. We have based our findings on the various analysis and interpretations of the patient’s condition through mathematical modeling. Although clinically speaking, anti-TNF-*α* has the strongest effect among the currently used drugs, some patients have not been showing any effects at all. In summary, prior to employing a biological treatment, a preliminary examination of the patient’s immune system is essential.

## Supporting information

S1 File (ZIP)
