# Supplementary material for: An immune therapy model for effective treatment on inflammatory bowel disease
Source: PLoS One. 2020 Sep 24;15(9):e0238918. doi: 10.1371/journal.pone.0238918 (PMC7514012; doi:10.1371/journal.pone.0238918)
Supplement: S1 File — (ZIP) [file pone.0238918.s001.zip › plos_anna_20200906_supporting information.pdf]

An immune therapy model for effective treatment on Inflammatory Bowel Disease

Anna Park[1,2], Sangil Kim[1,2], Il Hyo Jung[1,2], and Jong Hyuk Byun[1*,3]

1 Department of Mathematics, Pusan National University, Geumjeong-Gu, Busan 46241, South Korea
2 Finance·Fishery·Manufacture Industrial Mathematics Center on Big Data. Pusan National University, Busan, 46241, South Korea
3 Institute of Mathematical Sciences, Pusan National University, Busan, 46241, South Korea
* Corresponding Author: Jong Hyuk Byun (maticax@gmail.com)

Supporting information

S1 Table A. Fold changes of ratio of pro- to anti- inflammatory cytokines of patients compared with healthy individuals in each case before treatment.

Ratio of pro- to anti-inflammatory factors	Fold changes of patient level(%)			
	Case 1	Case 2	Case 3	Case 4
IFN-γ/IL-2	-99.76	-94.89	45.22	-95.82
IFN-γ/IL-4	-99.67	-97.96	-69.19	-98.93
IFN-γ/TFG-β	-97.73	-74.68	317.77	-90.70
IFN-γ /IL-10	-97.05	-70.61	-64.82	-88.57
IL-21/IL-2	-96.04	-94.60	220.97	-93.04
IL-21/IL-4	-94.58	-97.84	-31.87	-98.22
IL-21/TGF-β	-62.56	-73.21	823.72	-84.52
IL-21/IL-10	-51.23	-68.90	-22.22	-80.97
IL-6/IL-2	-93.82	-71.70	34.68	-63.08
IL-6/IL-4	-91.53	-88.66	-71.38	-90.54
IL-6/TGF-β	-41.52	40.41	288.08	-17.90
IL-6/IL-10	-23.81	62.99	-67.32	0.89
TNF-α/IL-2	775.45	1726.44	1187.96	1851.76
TNF-α/IL-4	1099.60	631.44	173.30	399.98
TNF-α/TGF-β	8180.64	8956.58	3605.33	4238.38
TNF-α/IL-10	10687.20	10412.96	211.98	5231.59
IL-12/IL-2	146.51	1031.40	2297.61	1374.97
IL-12/IL-4	237.78	353.34	409.89	277.95
IL-12/TGF-β	2231.62	5513.18	6812.96	3179.53
IL-12/IL-10	2937.40	6415.83	481.93	3930.34
T1/T2	9463.17	930.04	233.13	953.73
T1/Treg	106536.90	44555.27	5176.06	28644.98
T17/T2	-80.68	-97.16	-45.39	-96.25
T17/Treg	115.47	23.16	764.84	2.22

S1 Table B. Fold changes of ratios of pro- to anti- inflammatory factors of patients compared with healthy individuals using each anti-TNF-α and anti-IL-12 in Case 1.

Ratios of pro- to anti-inflammatory factors(Case 1)	Patient level	Anti-TNF-α	Anti-IL-12
IFN-γ /IL-2	-99.76	[-99.77, -99.76]	[-99.35, -97.95]
IFN-γ /IL-4	-99.67	[-99.76, -99.72]	[-99.98, -99.97]
IFN-γ /IL-TFG-β	-97.73	[-98.68, -98.14]	[-99.74, -99.61]
IFN-γ /IL-10	-97.05	[-98.07, -97.49]	[-99.70, -99.54]
IL-21/IL-2	-96.04	[-95.58, -94.71]	[-82.20, -21.18]
IL-21/IL-4	-94.58	[-94.92, -94.57]	[-99.38, -99.19]
IL-21/TGF-β	-62.56	[-71.27, -65.67]	[-90.75, -88.76]
IL-21/IL-10	-51.23	[-57.87, -53.80]	[-89.30, -86.76]
IL-6/IL-2	-93.82	[-94.66, -93.61]	[-35.38, 163.99]
IL-6/IL-4	-91.53	[-94.22, -92.54]	[-97.77, -97.14]
IL-6/TGF-β	-41.52	[-68.91, -50.87]	[-66.66, -60.83]
IL-6/IL-10	-23.81	[-54.32, -33.88]	[-61.49, -53.65]
TNF-α/IL-2	775.45	[157.91, 621.22]	[796.10, 867.17]
TNF-α/IL-4	1099.60	[195.28, 748.73]	[-91.77, -60.66]
TNF-α/TGF-β	8180.64	[1790.01, 5487.92]	[22.48, 441.18]
TNF-α/IL-10	10687.20	[2510.98, 7420.39]	[41.68, 539.94]
IL-12/IL-2	146.51	[112.88, 153.05]	[-76.29, 136.15]
IL-12/IL-4	237.78	[128.67, 196.30]	[-99.03, -89.63]
IL-12/TGF-β	2231.62	[1131.68, 1850.44]	[-86.38, 42.59]
IL-12/IL-10	2937.40	[1709.32, 2525.03]	[-83.97, 68.60]
T1/T2	9463.17	[6056.90, 7503.25]	[-77.92, 18.34]
T1/Treg	106536.90	[35686.98, 71502.11]	[11238.17, 25890.99]
T17/T2	-80.68	[-84.37, -82.10]	[-99.18, -98.89]
T17/Treg	115.47	[-7.09, 61.94]	[133.71, 535.75]

S1 Table C. Fold changes of ratios of pro- to anti- inflammatory factors of patients compared with healthy individuals using each anti-TNF-α and anti-IL-12 in Case 2.

Ratios of pro- to anti-inflammatory factors(Case 2)	Patient level	Anti-TNF-α	Anti-IL-12
IFN-γ /IL-2	-94.89	[-94.91, -94.90]	[-93.96,-90.32]
IFN-γ /IL-4	-97.96	[-98.33, -98.16]	[-99.98,-99.94]
IFN-γ /IL-TFG-β	-74.68	[-82.77, -77.89]	[-99.51,-98.49]
IFN-γ /IL-10	-70.61	[-79.35, -74.09]	[-99.44,-98.24]
IL-21/IL-2	-94.60	[-94.43, -93.88]	[-82.95,-24.71]
IL-21/IL-4	-97.84	[-98.06, -97.92]	[-99.87,-99.83]
IL-21/TGF-β	-73.21	[-79.35, -75.84]	[-96.43,-95.56]
IL-21/IL-10	-68.90	[-75.28, -71.68]	[-95.89,-94.85]
IL-6/IL-2	-71.70	[-75.21, -72.33]	[69.97, 579.81]
IL-6/IL-4	-88.66	[-91.66, -90.02]	[-98.77,-98.39]
IL-6/TGF-β	40.41	[-15.60, 19.95]	[-65.70,-57.17]
IL-6/IL-10	62.99	[1.12, 40.59]	[-60.61,-50.15]
TNF-α/IL-2	1726.44	[437.95, 1404.40]	[1776.60, 1958.14]
TNF-α/IL-4	631.44	[90.57, 442.77]	[-96.24,-82.38]
TNF-α/TGF-β	8956.58	[2163.37, 6422.47]	[4.62, 369.54]
TNF-α/IL-10	10412.96	[2572.42, 7544.67]	[20.27, 446.15]
IL-12/IL-2	1031.40	[884.47, 1001.04]	[-37.53, 521.86]
IL-12/IL-4	353.34	[230.22, 297.36]	[-99.46,-94.16]
IL-12/TGF-β	5513.18	[3245.35, 4673.64]	[-85.36, 55.55]
IL-12/IL-10	6415.83	[3908.36, 5495.10]	[-83.01, 80.93]
T1/T2	930.04	[730.05, 815.36]	[-96.65, -82.68]
T1/Treg	44555.27	[16904.44, 32189.08]	[5568.97, 12771.91]
T17/T2	-97.16	[-97.53, -97.28]	[-99.88, -99.84]
T17/Treg	23.16	[-47.41, -8.13]	[10.83, 205.00]

S1 Table D. Fold changes of ratios of pro- to anti- inflammatory factors of patients compared with healthy individuals using each anti-TNF-α and anti-IL-12 in Case 3.

Ratios of pro- to anti-inflammatory factors(Case 3)	Patient level	Anti-TNF-α	Anti-IL-12
IFN-γ /IL-2	45.22	[45.13, 46.45]	[43.86, 50.16]
IFN-γ /IL-4	-69.19	[-84.85, -77.46]	[-96.17,-76.00]
IFN-γ /TFG-β	317.77	[49.56, 205.14]	[150.72, 1087.22]
IFN-γ /IL-10	-64.82	[-83.74, -71.23]	[43.46, 256.81]
IL-21/IL-2	220.97	[322.52, 670.28]	[-75.85, 34.30]
IL-21/IL-4	-31.87	[-34.47, -18.31]	[-97.99,-94.47]
IL-21/TGF-β	823.72	[564.06, 798.96]	[52.00, 145.23]
IL-21/IL-10	-22.22	[-36.58, -1.48]	[-63.32, 52.80]
IL-6/IL-2	34.68	[46.19, 64.97]	[42.68, 275.97]
IL-6/IL-4	-71.38	[-82.81, -77.10]	[-90.65,-75.89]
IL-6/TGF-β	288.08	[64.92, 210.55]	[518.87, 1212.57]
IL-6/IL-10	-67.32	[-82.58, -70.72]	[97.99, 452.94]
TNF-α/IL-2	1187.96	[280.94, 964.77]	[1190.74, 1273.42]
TNF-α/IL-4	173.30	[-44.04, 64.91]	[-64.25, 114.50]
TNF-α/TGF-β	3605.33	[406.64, 2138.19]	[2240.02, 10550.84]
TNF-α/IL-10	211.98	[-47.83, 111.03]	[1187.54, 3154.03]
IL-12/IL-2	2297.61	[2003.06, 2524.89]	[-51.98, 386.65]
IL-12/IL-4	409.89	[151.01, 296.26]	[-93.08,-20.38]
IL-12/TGF-β	6812.96	[2199.74, 5275.32]	[249.03, 3886.67]
IL-12/IL-10	481.93	[139.89, 406.83]	[-33.32, 1082.73]
T1/T2	233.13	[68.28, 143.62]	[-60.93, 143.65]
T1/Treg	5176.06	[1704.30, 3751.30]	[7648.77, 29794.51]
T17/T2	-45.39	[-47.53, -31.78]	[-98.49, -95.74]
T17/Treg	764.84	[489.24, 731.69]	[170.67, 635.17]

S1 Table E. Fold changes of ratios of pro- to anti- inflammatory factors of patients compared with healthy individuals using each anti-TNF-α and anti-IL-12 in Case 4.

Ratio of pro- to anti-inflammatory factors(Case 4)	Patient level	Anti-TNF-α	Anti-IL-12
IFN-γ /IL-2	-95.82	[-95.84, -95.82]	[-94.43, -89.12]
IFN-γ /IL-4	-98.93	[-99.10, -99.00]	[-99.97, -99.93]
IFN-γ /TFG-β	-90.70	[-93.09, -91.60]	[-99.63, -99.11]
IFN-γ /IL-10	-88.57	[-91.09, -89.54]	[-99.58, -98.97]
IL-21/IL-2	-93.04	[-93.21, -92.98]	[-71.37, 26.58]
IL-21/IL-4	-98.22	[-98.50, -98.36]	[-99.68, -99.62]
IL-21/TGF-β	-84.52	[-88.38, -86.19]	[-95.94, -95.29]
IL-21/IL-10	-80.97	[-85.03, -82.80]	[-95.34, -94.55]
IL-6/IL-2	-63.08	[-68.26, -63.76]	[140.99, 892.44]
IL-6/IL-4	-90.54	[-92.91, -91.37]	[-97.40, -96.88]
IL-6/TGF-β	-17.90	[-46.33, -27.15]	[-66.77, -61.26]
IL-6/IL-10	0.89	[-30.65, -9.28]	[-61.88, -55.07]
TNF-α/IL-2	1851.76	[474.82, 1507.35]	[1924.15, 2191.68]
TNF-α/IL-4	399.98	[35.31, 282.74]	[-93.95, -73.91]
TNF-α/TGF-β	4238.38	[1036.92, 3131.15]	[-22.91, 223.89]
TNF-α/IL-10	5231.59	[1319.41, 3923.66]	[-11.49, 275.51]
IL-12/IL-2	1374.97	[1170.11, 1343.56]	[-11.51, 781.31]
IL-12/IL-4	277.95	[182.24, 243.78]	[-98.92, -88.64]
IL-12/TGF-β	3179.53	[2038.86, 2801.90]	[-86.56, 40.99]
IL-12/IL-10	3930.34	[2663.53, 3513.74]	[-84.45, 63.46]
T1/T2	953.73	[778.05, 870.98]	[-91.75, -59.34]
T1/Treg	28644.98	[14127.57, 22905.01]	[6665.79, 14848.79]
T17/T2	-96.25	[-96.90, -96.59]	[-99.49, -99.39]
T17/Treg	2.22	[-49.21, -19.31]	[115.62, 530.19]

S1 Table F. Fold changes of ratios of pro- to anti- inflammatory factors of patients compared with healthy individuals in administration IL-10.

Ratio of pro- to anti-inflammatory facotrs	Case 1	Case 2	Case 3	Case 4
IFN-γ /IL-2	[-99.76, -99.75]	[-94.92,-94.81]	[45.19, 46.67]	[-95.85,-95.74]
IFN-γ /IL-4	[-99.96, -99.89]	[-99.66,-99.09]	[-82.68,-74.88]	[-99.76,-99.38]
IFN-γ /TFG-β	[-99.85, -99.44]	[-96.54,-88.86]	[162.04, 265.86]	[-98.45,-95.19]
IFN-γ /IL-10	[-99.95, -99.55]	[-99.21,-93.78]	[-81.75,-68.73]	[-99.66,-97.20]
IL-21/IL-2	[-54.30, 127.19]	[-90.79,-76.68]	[249.10, 389.91]	[-87.88,-68.47]
IL-21/IL-4	[-80.50, -65.62]	[-98.67,-98.25]	[-45.31,-39.42]	[-98.28,-98.13]
IL-21/TGF-β	[-29.30, 60.93]	[-84.84,-79.82]	[730.59, 800.37]	[-89.27,-85.95]
IL-21/IL-10	[-86.63, -9.55]	[-98.35,-88.72]	[-53.23,-24.82]	[-98.81,-91.84]
IL-6/IL-2	[-93.61, -92.12]	[-73.18,-69.14]	[44.55, 73.07]	[-66.74,-61.43]
IL-6/IL-4	[-98.86, -96.95]	[-98.14,-95.19]	[-79.57,-74.99]	[-97.94,-94.97]
IL-6/TGF-β	[-95.65, -85.16]	[-80.94,-41.03]	[210.85, 264.23]	[-86.77,-61.11]
IL-6/IL-10	[-98.48, -88.13]	[-95.49,-67.05]	[-80.63, -68.87]	[-97.02,-77.42]
TNF-α/IL-2	[775.46, 776.02]	[1725.81, 1727.73]	[1191.47, 1201.63]	[1850.36, 1852.77]
TNF-α/IL-4	[28.41, 317.95]	[21.13, 226.27]	[55.03, 123.40]	[13.20, 192.61]
TNF-α/TGF-β	[410.93, 1932.98]	[1136.12, 3900.78]	[2242.79, 3154.16]	[622.43, 2160.94]
TNF-α/IL-10	[89.73, 1526.38]	[180.86, 2135.44]	[62.24, 178.11]	[57.49, 1212.88]
IL-12/IL-2	[58.68, 130.04]	[593.70, 868.07]	[2016.08, 2592.91]	[772.56, 1112.97]
IL-12/IL-4	[-70.64, 8.72]	[-49.93, 72.92]	[208.47, 337.77]	[-45.51, 81.93]
IL-12/TGF-β	[8.59, 428.88]	[394.86, 2020.71]	[4268.87, 6277.21]	[240.97, 1305.77]
IL-12/IL-10	[-63.65, 323.22]	[8.32, 1085.22]	[169.75, 445.03]	[-28.17, 716.45]
T1/T2	[378.40, 1858.12]	[35.28, 287.60]	[86.27, 169.80]	[79.50, 411.89]
T1/Treg	[2172.05, 11037.46]	[2183.81, 10368.87]	[3325.21, 4566.09]	[1807.74, 8238.60]
T17/T2	[-55.12, -44.36]	[-98.64,-97.96]	[-56.71, -50.96]	[-97.35,-96.60]
T17/Treg	[44.49, 248.66]	[-73.10,-50.82]	[694.96, 776.43]	[-70.93,-48.45]
